# Supplementary material for: Analysis of PPARγ Signaling Activity in Psoriasis
Source: Int J Mol Sci. 2021 Aug 10;22(16):8603. doi: 10.3390/ijms22168603 (PMC8395241; doi:10.3390/ijms22168603)
Supplement: Supplementary file 1 [file ijms-22-08603-s001.zip › Supplemental materials_Analysis of PPARg signaling activity in psoriasis/Pathway models/Models images and html files/PPARG inhibits phenotypes assotiated with psoriasis/135303.html]

insulin resistance


# Disease insulin resistance

|  |  |
| --- | --- |
| URN | urn:agi-meshdis:Insulin%20Resistance |
| Total Entities | 4 |
| Connectivity | 8465 |
| Name | insulin resistance |

---

|  |  |
| --- | --- |
| ChildConcepts | extreme insulin resistance syndrome |
|  | insulin unresponsiveness |
|  | insulin insensitivity |
|  | SHORT syndrome |

---

|  |  |
| --- | --- |
| Pathway | Familial Partial Lipodystrophy Type 4 Progression (Hypothesis) |
|  | Donohue Syndrome Progression (Hypothesis) |
|  | Familial Partial Lipodystrophy Type 3 Progression (Hypothesis) |
|  | Familial Partial Lipodystrophy Type 2 Progression (Hypothesis) |
|  | Berardinelli-Seip Syndrome Progression (Hypothesis) |
|  | Proteins Involved in Insulin Resistance |
|  | PPARG inhibits phenotypes assotiated with psoriasis |
|  | efects selected |
|  | Neighbors of insulin resistance |

---

|  |  |
| --- | --- |
| MedScan ID | 9012899 |

---

|  |  |
| --- | --- |
| Alias | insulin resistances |
|  | insulin resistance |

---

|  |  |
| --- | --- |
| MeSH ID | D007333 |
|  | M0011432 |

---

|  |  |
| --- | --- |
| MeSH Heading | Insulin Resistance |

---
